# New Graduate Nurses' Clinical Competence, Clinical Stress, and Intention to Leave: A Longitudinal Study in Taiwan

**DOI:** 10.1155/2014/748389

**Published:** 2014-01-29

**Authors:** Ching-Yu Cheng, Hsiu-Min Tsai, Chia-Hao Chang, Shwu-Ru Liou

**Affiliations:** ^1^Chang Gung University of Science and Technology, 2 Chiapu Road, West Sec., Putz, Chiayi 613, Taiwan; ^2^Chang Gung University of Science and Technology, 261 Wen-hwa 1st Road, Kwei-shan, Taoyuan 333, Taiwan

## Abstract

This longitudinal research study aimed to develop a pregraduation clinical training program for nursing students before graduation and evaluate its effect on students' self-perceived clinical competence, clinical stress, and intention to leave current job. A sample of 198 students returned the questionnaires before and after the program. They were followed up at 3, 6, and 12 months after graduation. Results showed that posttest clinical competence was significantly higher than pretest competence, positively related to clinical competence at 3 and 12 months, and negatively related to clinical stress at 3 months. The clinical competence at 3 months was positively related to clinical competence at 6 and 12 months, and clinical competence at 6 months was related to intention to leave at 12 months. Intention to leave at 6 months was positively related to intention to leave at 3 and 12 months. Clinical stress at 3 months was positively related to clinical stress at 6 and 12 months, but not related to intention to leave at any time points. The training program improved students' clinical competence. The stressful time that was correlated with new graduate nurses' intention to leave their job was between the sixth and twelfth months after employment.

## 1. Introduction 

Nurse shortage has become a worldwide issue. Using the United States as an example, the shortage of hospital registered nurses will reach 340,000 nurses by the year 2020 [1]. Nurse turnover creates staffing shortages that increase the work demands placed on the organization's remaining nurses [2]. According to a survey reported by the International Council of Nursing [3], the overall nurse turnover rate within the first year of employment in Taiwan was about 28%. Regionally, the Taipei City Nursing Board [4] in Taiwan reported that the turnover rate of new graduate nurses during the first year of employment was 57.7% and turnover was at its peak of 32.1% within the first 3 months of employment. The turnover rate of new graduate nurses in Taiwan was 2-3 times higher than the rates in other Asian countries such as Hong Kong, Japan, Korea, and Singapore [3]. High turnover rates and short periods of employment among new graduate nurses are costly for healthcare organizations. The cost could be as high as USD $41,000 to recruit a new graduate with less than 1 year of employment experience [5]. In addition, the implicit costs include preceptor exhaustion and decreased unit morale; one or two instances of turnover might trigger additional nurses' turnover [2]. With those reasons, recruitment and retention of new graduate nurses have become the first priority for many healthcare facilities to solve the problem of nurse shortage.

Although recruiting new graduate nurses was identified as important, according to Benner's Novice to Expert Theory [6], new graduate nurses are limited in technological skills and lacking clinical experience and thus unprepared to begin practice. Specifically, only 10% of healthcare administrators considered new graduate nurses ready to perform safe and effective patient care [7]. New graduate nurses therefore require a transition period before becoming a competent clinical nurse [8]. Many hospitals have developed programs to facilitate new graduate nurses' transition from student to staff nurse. These programs help to improve new graduate nurses' clinical competence during the first year of employment, which in turn helps to ease the problem of high turnover. Whether successful or not, these programs share a common characteristic: they were conducted in the orientation period after new graduate nurses enter the new unit. For many new graduate nurses, the orientation period is arduous. They experience stress as they acquire competence in performing patient care, take on more responsibilities, learn about the nurse role, and more importantly strive to meet role expectations during this time period [9]. If new graduate nurses attend a training program before graduation and gain clinical competence at least in part before beginning their first job, they may attain their new role faster and smoother.

## 2. Background and Significance

Kramer [10] coined the phrase “reality shock” to describe the beginning work experience of new nurses. She defined reality shock as “the shock-like reaction that occurs when an individual who has been reared and educated in that subculture of nursing that is promulgated by schools of nursing suddenly discovers that nursing as practiced in the world of work is not the same—it does not operate on the same principles” (page 291) [10]. Reality shock consists of four phases that are experienced sequentially by new graduates. Phase one is the honeymoon phase, characterized by excitement and euphoria about having a real nursing job and about getting paid for the work. Phase two is the shock phase, which begins when the new nurses discover that the reality of the clinical work does not match their expectations based on what they were taught. In phase two new graduate nurses' goals and hospitals' expectations may not be consistent because of the nurses' inexperience or the strict nature of the work environment. Phase three is recovery, in which new graduate nurses begin to look at their work situation objectively and stress and anxiety decrease. Phase four is resolution which can be either positive or negative—the new graduate nurses either learn new ways to cope effectively with their new role or they reject the role and continue to experience symptoms of stress and burnout.

This study focused on Kramer's honeymoon and shock phases because these are regarded as the most difficult phases for new graduate nurses [10]. The description of new graduates' experiences in the shock phase is consistent with Benner's description of new graduate nurses as novices [6], in her Novice to Expert Theory. Novices have textbook knowledge but lack the clinical experience to apply the knowledge to practice. Current nursing education is mostly conducted in a classroom setting. Student nurses get their clinical experiences in multiple clinical sites rather than the setting where they will work after graduation. Therefore, when new graduate nurses move from the school environment to the work setting, they experience conflicts due to the inconsistent roles, values, and nursing abilities they learned from the school and various clinical sites. Not surprisingly, phase two of reality shock is described as experiencing conflicts, pressures, and dissatisfaction that may lead to intention to quit the job.

A substantial body of research findings indicates that the transition of new graduate nurses from an academic program to a clinical setting is a stressful time period [11]. New graduates have to quickly accommodate themselves to new roles and responsibilities, overcome the differences between the theoretical orientation of their education and the practical focus of clinical practice, and integrate into an environment that emphasizes teamwork, all in the initial 3 months of transition from student to staff nurse [12, 13]. Stresses that new graduates face are related to a lack of competence including skill performance; responding appropriately to emergencies; admitting new patients; communication with physicians; developing relationships with colleagues, patients, and families; managing workload demands; organizing and prioritizing duties; doing shift reports; and adjusting to different shifts [14]. New graduate nurses may leave their job if these transitional stresses are not resolved during this period [11].

According to Casey et al. [15] new graduate nurses need at least 1 year to feel comfortable and confident to work in a new environment, and from three to twelve months is the most difficult period of adjustment. Other studies report that the first three to six months of employment is the most stressful period, a critical stage when new graduate nurses form their intention to leave their current job [12, 16]. The turnover rate for nurses in their first year employment is about 30% and it is about 57% by the second year in the United States [17, 18]. Retaining new graduate nurses during the first year of employment, therefore, is imperative and challenging for hospital managers.

Researchers [19] pointed out the common reason for new graduate nurses to consider leaving their job within the first year of employment is because they do not feel they fit in the work environment. An estimated 33–61% of new nurses in North America are planning to change their job or leave the nursing profession within their first year of clinical practice [8, 17]. The intention to leave is especially important since it is a significant predictor of turnover behavior [20]. Researchers have concluded that stress is significantly related to intention to leave among healthcare providers. Even though many healthcare providers find their work satisfying, they often think about leaving their jobs because of job stress [21, 22]. Therefore, helping new graduates to overcome conflicts and stress is imperative since that can promote their retention and lower the turnover rate [23, 24].

**Figure 1:**
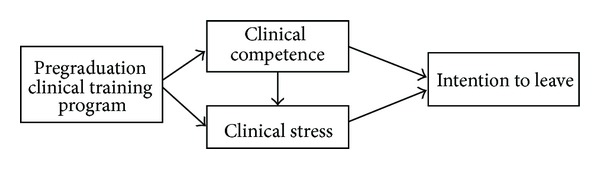
Study conceptual framework.

Studies have focused on the relationship between clinical stress and intention to leave among new graduate nurses, but little attention has been paid to the relationships between clinical competence, clinical stress, and intention to leave all together in a longitudinal study. Clearly, the new employment orientation period encompasses substantial learning and is a stressful time for new graduate nurses. Therefore, the purpose of this study was to develop a pregraduation clinical training program for senior nursing students in their last semester before graduation and evaluate the effects of this program empirically and longitudinally on students' self-perceived clinical competence, clinical stress, and intention to leave their current job. The conceptual framework guiding this study is illustrated in Figure 1. The research questions guiding this study are as follows: (1) What is the relationship between clinical competence, clinical stress, and intention to leave longitudinally? (2) What is the effect of the pregraduation clinical training program on clinical competence among new graduate nurses in their first year of employment? (3) Is there any difference in clinical competence, clinical stress, and intention to leave between students who remain in the hospital where they completed the pregraduation clinical training program and students who do not remain?

## 3. Methods

### 3.1. Development of the Pregraduation Clinical Training Program

The pregraduation clinical training program was designed to bridge the gap between student and professional nurse roles and was implemented for the first time at our school when this study started. This program was a three-credit hour course required for graduation. Participants were students in a two-year baccalaureate degree nursing program (RN-to-BSN). They had previously completed a five-year diploma nursing program and were licensed to practice nursing. However, these students directly entered the RN-to-BSN program as full-time students without any nursing work experience. The aim of the program was to provide clinical placements at hospitals where students wanted to get jobs following graduation. The training program thus served to facilitate enhanced competence development and also helped nursing students become familiar with their future work place while they were still students. This familiarity might reduce new graduates' experience of reality shock and prevent turnover, avoiding costs for hospitals. Therefore, the school faculty had meetings with students first to determine their preferred hospitals for practice. However, based on the school's regulation that all hospitals where students had clinical practicum must pass the evaluation by the Department of Health in Taiwan, not all students' preferred hospitals could be selected. We then visited each of those students' preferred hospitals and explained the purpose and content of the pregraduation clinical training program with hospital administrators.

The course required students to practice as a registered nurse under the supervision of a hospital registered nurse preceptor for a total of 240 hours within six weeks. Each student was assigned with a preceptor during the program and scheduled to work the same schedule with their preceptor, which had to include at least two different kinds of shifts. To qualify as preceptors, nurses at the clinical facilities had to have passed a preceptor training program held by their facility. During the pregraduation clinical training program, the school faculty served as external mentors, facilitating student nurses' clinical learning and acting as a coordinator between the school and the clinical facilities. Faculty conducted four-hour discussion meetings biweekly with all the students in the program. These discussions provided opportunities for students to share experiences with each other and served as a channel for communication between students, faculty, and clinical facilities.

### 3.2. Research Design

The study was a longitudinal design. All students received the designed training program. Data was collected before implementation of the training program (pretest), after the conclusion of the training program (posttest), and at three, six and twelve months after students graduated and began their first year of employment.

### 3.3. Research Sampling and Setting

Two hundred six pregraduates students enrolled in the final semester of the two-year RN-to-BSN program in the investigators' school were contacted and invited to participate in the study and 198 students completed the pretest and posttest questionnaires, and 111, 101, and 75 new graduate nurses returned questionnaires in the third, sixth, and twelfth months after they graduated and began their first job. The study response rate was 96.12% in the pretest and posttest and was 53.88%, 49.03%, and 36.41% in the third, sixth, and twelfth months after graduation. The participants completed all questionnaires at places they felt comfortable at.

### 3.4. Procedure and Ethical Consideration

The data collection started after a local research ethics committee in Taiwan approved the study protocol. Following the approval, we held a meeting with the students to explain the program and the research study, including the study purpose, procedures, participants' rights, and confidentiality. This information was also included in the cover letter that was sent to the participants along with a set of questionnaires and a self-addressed-and-stamped envelope before each data collection time point. Although the participants were not required to sign and return a consent form, finishing and returning the questionnaires indicated participants' agreement to participate in this study. For participants' convenience, they could choose to fill out the questionnaires either in a paper-and-pencil form or in an electronic form. If the participants chose to use the paper-and-pencil form, they had to mail back the completed questionnaires using the provided envelope. If the participants chose to use email, they had to reply to the invitation email sent by the research team and attach the completed questionnaires to their emails. Emails that could identify the participants were deleted after the electronic completed questionnaire was saved in a secured computer that was protected with passwords known only to the primary investigator.

### 3.5. Instruments

*The Clinical Competence Questionnaire (CCQ)*. The CCQ is an instrument developed by Liou and Cheng [25] to evaluate new graduate nurses' level of clinical competence in the first year following their graduation from nursing school. The CCQ is a 47-item 5-point Likert-type scale. Each item score ranges from 1 (do not have a clue) to 5 (know in theory, competent in practice without any supervision). A higher score indicates higher competence. Validity of the CCQ was evidenced by factor analysis that resulted in two factors: 16 items of nursing professional behaviors and 31 items of skill competencies. The nursing professional behavior items explained 59.6% of the variation in competence in nursing professional behaviors; the skill competencies items explained 70.72% of the variation in competence in clinical skills [25]. Cronbach's alpha for the entire CCQ was .97 and was .95 for both the nursing professional behaviors and the skill competencies subscales in this study. 

*The Clinical Stress Scale (CSS).* The CSS is an instrument developed by the investigators based on the literature reviews to evaluate new graduate nurses' level of clinical stress. The CSS is a 46-item 5-point Likert-type scale (ranges from 0 to 4). Sample questions are “I have knowledge and skills to implement nursing care” and “I can identify and apply related work resources.” A higher score indicates a higher level of stress. Validity of the scale was confirmed by exploratory factor analysis that showed 76.28% of the variation in clinical stress could be explained. Cronbach's alpha of the CSS was .95 in this study.

*The Anticipated Turnover Scale (ATS).*   The new graduates' intention to leave their current job was measured by the Anticipated Turnover Scale (ATS), which was developed by Hinshaw et al. [26]. The purpose of the ATS is to measure individuals' perception or opinions about voluntarily leaving their present job. The ATS is a 12-item self-report instrument using 5-point Likert-type scale that ranges from 5 (strongly agree) to 1 (strongly disagree). A higher score indicates a stronger intention to leave the job. The Cronbach's alpha of the ATS was .84 and construct validity was supported by factor analysis in the scale development study [26]. In this study, the Cronbach's alpha of the ATS in the current study was .90. Validity was confirmed by exploratory factor analysis that demonstrated the 70.24% of the variation in intention to leave could be explained.

### 3.6. Data Analysis

All data were entered and analyzed using SPSS version 17.0. All tests were 2 sided and *P* values less than .05 were considered statistically significant. All students in this study received the designed pregraduation clinical training program and therefore the program was not treated as an independent variable. Participants' demographic information and levels of major measured variables were analyzed by descriptive statistics. Normality of the measured variables was tested using the Shapiro-Wilk test. Results showed that clinical competence at all measuring time points and intention to leave in the third and twelfth months were not normally distributed. Therefore, Spearman's correlation was used to examine relationships between measured variables that were not normally distributed whereas Pearson correlation was used for normally distributed variables. The Generalized Estimating Equations analysis introduced by Liang and Zeger [27] was used to analyze longitudinal and repeated measured data for clinical competence to understand changes across the five data collection points (pretest, posttest, and three, six, and twelve months after graduation). The main advantage of Generalized Estimating Equations analysis resides in the robust estimation of parameters' standard errors, even when the correlation structure is misspecified. Users of Generalized Estimating Equations analysis can be more confident in their statistical conclusions regarding data that arise from a longitudinal research design [27].

## 4. Results

### 4.1. Descriptive Statistics

All participants were females with an average age of 23 years. Upon the completion of the pregraduation clinical training program, only 3.9% of students felt dissatisfied with their performance during the training program. Almost all students were satisfied with the school's arrangement of the program (98.5%), 96.5% felt that the hours of the training program were appropriate, and 99% were satisfied with the preceptorship during the practice.

As shown in Table 1, after graduating from the school, more new graduate nurses worked in nonmedical centers, worked in medical-surgical units, stayed in the hospital or unit where they had the training program, were either satisfied or unsatisfied with their clinical performance, were either confident or unconfident in their professional competence. While 30.9% of new graduate nurses agreed that the pregraduation clinical training program was helpful in reducing their clinical job stress, 48.7% agreed that the program was helpful to their clinical competence, and 67.0% expressed the necessity of continuing the program in the future.

### 4.2. Clinical Competence within the First Year of Employment

Participants rated their clinical competence as high and their clinical stress and intention to leave their job as medium low across all data collection time points (Table 2). The Generalized Estimating Equations analysis (Table 2) revealed a significantly higher posttest clinical competence (postcompetence) score compared to the pretest clinical competence score. The clinical competence score at 3 months (3-month competence) was lower than the post-competence; however, the difference was not significant. The clinical competence scores at 6 months (6-month competence) and 12 months (12-month competence) were higher than the post-competence scores.

Participants experienced median high level of clinical stress within one year of employment but did not have high intention to leave their current job (Table 2). Compared to clinical stress in the third month, participants' clinical stress in the sixth and twelfth months did not show difference. Compared to intention to leave in the third month, participants' intention to leave in the sixth and twelfth months did not show difference.

### 4.3. The Relationships between Clinical Competence, Clinical Stress, and Intention to Leave

Table 3 depicts the relationships between clinical competence, clinical stress, and intention to leave. The post-competence was positively correlated with 3-month competence (*r* = .35,  *P* < .001) and 12-month competence (*r* = .33,  *P* < .001) but not 6-month competence. The 3-month competence score was positively correlated with 6-month competence (*r* = .29,  *P* < .001) and 12-month competence (*r* = .61,  *P* < .001), and 6-month competence was positively correlated with 12-month-competence (*r* = .40,  *P* < .001). The post-competence was negatively correlated with clinical stress only at three months (*r* = −.21,  *P* < .05), but was not correlated with intention to leave at any time point. The 3-month-competence was negatively correlated with clinical stress at three months (*r* = −.20,  *P* < .05) but was not correlated with intention to leave at any time point. The 6-month competence was correlated with intention to leave at twelve months (*r* = .25,  *P* < .05). The clinical stress at three months was positively correlated with clinical stress at six months (*r* = .59, *P* < .001) and clinical stress at twelve months (*r* = .54,  *P* < .001) but was not related to intention to leave at any time point. The clinical stress at six months was positively correlated with clinical stress at twelve months (*r* = .64,  *P* < .001) and intention to leave at six months (*r* = .36,  *P* < .001). The intention to leave at three, six, and twelve months was positively intercorrelated (*r* ranged from .40 to .59, *P* < .001). The intention to leave at three months was not related to clinical competence and clinical stress at any time point.

### 4.4. Comparison of Measured Variables by Variable of Remaining in the Training Hospital

Scores of clinical competence (*n* = 41, M = 195.80, SD = 25.25), clinical stress (M = 93.24, SD = 25.16), and intention to leave (M = 32.12, SD = 8.55) in the third month of the participants who remained in the hospital where they completed the pregraduation clinical training program and the scores of those who did not remain (*n* = 75, M = 189.92, SD = 31.91 for clinical competence; M = 94.87, SD = 33.06 for clinical stress; and M = 32.07, SD = 7.53) did not show difference (*F* = 1.04,  *P* = .31; *F* = .08,  *P* = .79;  *F* = .001,  *P* = .97, resp.).

## 5. Discussion

Study results reveal that, in general, the pregraduation clinical training program was effective in increasing students' clinical competence. This result is consistent with the other research findings that developing special programs to bridge the gap between academic education and clinical practice can improve new graduate nurses' clinical competence [23, 28, 29]. Pattern of students' clinical competence during one year after their employment was worth noting. The present study found that participants' clinical competence though significantly increased right after the program but slightly decreased in the third month after the students began their first job. Students' clinical competence gradually increased in the sixth month and reached a significantly higher score at one year compared to immediately after completing the training program. This result suggested that the time frame between the third and sixth months of employment was the crucial time period for new graduate nurses' acquisition of clinical competencies. This pattern provides support for Kramer's Reality Shock Theory [10], which points out that reality shock occurs when the new graduate nurses discover that the reality of the clinical work is beyond their expectations. This finding also provides support for Benner's Novice to Expert Theory [6], which proposes that new graduate nurses are novice nurses who have textbook knowledge but lack the clinical experience to apply the knowledge to clinical practice. In these circumstances the expectations of new graduate nurses and expectations of hospital employers may not be consistent. This inconsistency leads to conflicts, which may induce stress, which could be seen in the study results. In this study, new graduate nurses' clinical competence scores increased to a significantly higher score at one year compared to their scores immediately after completing the training program. This result confirms the findings of earlier studies that new graduate nurses need to have at least 12 months to feel comfortable and confident to work in a new environment [11, 15].

This study also found that students' clinical competence after the pregraduation clinical training program and in the third month after employment was negatively correlated with clinical stress in the third month after employment. During this period, new nurses may have difficulties in adjusting to their new roles and may experience an increase in stress associated with job expectations and lower clinical competence. In addition, new graduate nurses experienced median high level of stress and the stress remained about the same level within one year of employment. This result is similar to results of previous studies suggesting that the time frame between three and six months was the most stressful time for new graduate nurses and that caused new graduate nurses to have strong intention to leave their current job [16, 30]. However, this study did not find relationships between clinical competence, clinical stress, and intention to leave except that clinical stress was related to intention to leave in the sixth month and clinical competence in the sixth month was related to intention to leave in the twelfth month. This might imply that if the work environment cannot satisfy the new graduate nurses or brings stress to the new graduate nurses after six months of their employment, the new graduate nurses who are more clinically competent are more prone to have plans to leave their job.

The nonsignificant relationship between clinical stress, competence, and intention to leave could possibly be explained by other reasons. The new graduate nurses had worked for only three months and might have rationalized their lower clinical competence and higher stress as unfamiliarity with the work environment. They may have considered that it was too soon to feel completely competent and thus were not feeling at that time any intention to leave their current job. These results also confirmed the Reality Shock Theory that new nurses in the phase one honeymoon stage focused more on the excitement and euphoria of having a real nursing job and about getting paid for the work.

## 6. Limitation and Recommendation

The results of this study presented evidence that new graduate nurses benefited from the structured pregraduation clinical training program, including an improvement in clinical competence. However, generalisability of the study's findings is limited because only students studying in the two-year RN-to-BSN nursing program in one university were included in the sample. To increase the generalisability of the results, it is recommended that future investigations include nursing students in other nursing programs such as the traditional four-year Bachelor's program or five-year diploma program. This study did not have a comparison group who completed a regular clinical practicum, because we wanted to let all students equally have the chance to participate in the program. Although this study found that new graduate nurses who participated in the pregraduation clinical training program had significantly higher posttest clinical competence score compared to pretest, the strength of the conclusion drawn—that the program results in significant increase in clinical competence—is limited by the lack of a comparison group in this study. Therefore, it is recommended that future research includes a comparison group that does not participate in the pregraduation clinical training program to increase the power of the conclusion. Qualitative interviews of the unit managers may also provide valuable insight, and it is suggested that future studies include these interviews to support the quantitative findings by furnishing an in-depth and alternative perspective of the situation.

## 7. Conclusion

This study provides meaningful information to nurse educators and healthcare administrators. The results revealed that the pregraduation clinical training program started nurse students' learning about how to be a professional nurse before they left school, was effective in promoting students' clinical competence, and was conducive to nurse students' adaptation to their clinical jobs following graduation. New graduate nurses' clinical competence remained higher after the program than before the program. Although new graduate nurses' clinical competence dropped insignificantly in the third month, it increased in the sixth and twelfth months. There are two implications for this result. First, the pregraduation clinical training program might play an important role to subdue a certain degree of reality shock during these months and help new graduate nurses regain higher levels of competence quickly. Second, the period from postgraduation to the sixth month is a critical time for new graduate nurses to reinforce clinical competence. Hospitals that can provide a supportive environment and training that meets new graduate nurses' needs during this period can help nurses establish higher levels of competence and decrease the subsequent clinical pressure. Finally, this study revealed that the most stressful time was the first three months, but the stressful time that was correlated with new graduate nurses' intention to leave their job was between the sixth and twelfth months after employment. This finding has significant implications for healthcare administrators. The golden time for hospitals to promote new graduate nurses retention must start at the beginning of employment, or this high level of stress may be accumulated over time and, finally, causes nurses to leave their jobs. More importantly, hospital administrators should not view new graduate nurses as an immediate solution to the nurse shortage. Instead, a long-term strategy should be applied, aiming at cultivating the professional development of new graduate nurses over at least twelve months. This may be the golden rule for successful retention of new graduate nurses.

## Figures and Tables

**Table 1:** Descriptive information during one year of employment.

	3 months *n* (%)	6 months *n* (%)	12 months *n* (%)
Hospital type			
Medical center	46 (42.2)	43 (43.0)	33 (44.6)
Nonmedical center	63 (57.8)	57 (57.0)	41 (55.4)
Unit			
Medical-surgical	63 (57.8)	54 (54.5)	38 (50.7)
ICU/ER	36 (33.0)	35 (35.4)	30 (40.0)
Maternity/pediatrics/psychology	7 (6.4)	8 (8.1)	5 (6.7)
Others	3 (2.8)	2 (2.0)	2 (2.7)
Stayed in the hospital where they have the training program			
No	70 (63.6)	69 (68.3)	50 (67.6)
Yes	40 (36.4)	32 (31.7)	24 (32.4)
Stayed on the unit where they have the training program			
No	96 (88.9)	87 (87.0)	62 (82.7)
Yes	12 (11.1)	13 (13.0)	13 (17.3)
Work shifts			
8 hours	99 (91.7)	89 (88.1)	67 (91.8)
12 hours	3 (2.8)	4 (4.0)	2 (2.7)
Both 8 hours and 12 hours	6 (5.6)	8 (7.9)	4 (5.5)
Satisfaction with clinical performance			
Satisfied	24 (21.8)	30 (29.7)	32 (43.3)
Neutral	65 (59.1)	64 (63.4)	39 (52.7)
Unsatisfied	21 (19.1)	7 (6.9)	3 (4.0)
Confident in professional competence			
Confident	19 (17.3)	33 (32.7)	31 (41.4)
Neutral	70 (63.6)	61 (60.4)	41 (54.7)
Not confident	21 (19.1)	7 (6.9)	3 (4.1)
Agree that the program helped in reducing job stress			
Agree	34 (30.9)	—	—
Neutral	44 (40.0)	—	—
Disagree	32 (29.1)	—	—
Agree that the program helped in clinical work			
Agree	53 (48.7)	—	—
Neutral	41 (37.6)	—	—
Disagree	15 (13.8)	—	—
Necessity of continuing the program in the future			
Agree	73 (67.0)	—	—
Neutral	31 (28.4)	—	—
Disagree	5 (4.6)	—	—

**Table 2:** Comparison of measured variables by time.

	Possible range	Study range	M ± SD	Wald *X* ^2^	*P*
Clinical competence					
Pretest	47–235	53–235	189.0 ± 27.72	7.45	.01
Posttest (referent)	47–235	76–235	195.3 ± 20.94	—	—
Third month	47–235	75–235	193.0 ± 30.25	1.28	.26
Sixth month	47–235	51–235	198.6 ± 30.82	7.56	.01
Twelfth month	47–235	85–235	209.1 ± 24.38	36.25	<.001
Clinical stress					
Third month (referent)	0–184	16–157	94.5 ± 29.38	—	—
Sixth month	0–184	27–163	90.49 ± 27.87	1.71	.19
Twelfth month	0–184	23–153	89.8 ± 29.83	1.94	.16
Intention to leave					
Third month (referent)	12–60	16–55	32.4 ± 8.11	—	—
Sixth month	12–60	16–48	32.4 ± 6.43	.02	.88
Twelfth month	12–60	17–56	33.4 ± 8.30	2.10	.15

**Table 3:** Relationships between measured variables.

	preCC	postCC	CC3	CC6	CC12	CS3	CS6	CS12	ITL3	ITL6
preCC	1.00									
postCC	.14	1.00								
CC3	.14	.35**	1.00							
CC6	.08	.19	.29**	1.00						
CC12	.12	.33**	.61**	.40**	1.00					
CS3	−.01	−.21*	−.20*	−.09	−.01	1.00				
CS6	.07	−.17	−.06	−.01	−.08	.59**	1.00			
CS12	.06	−.03	.00	−.19	−.16	.54**	.64**	1.00		
ITL3	.05	−.02	.03	.05	.02	.17	.05	.17	1.00	
ITL6	−.04	−.02	.09	.14	.02	.22	.36**	.25	.47**	1.00
ITL12	.03	.08	−.02	.25*	.03	.04	.03	.05	.40**	.59**

preCC: pretest clinical competence; postCC: posttest clinical competence; CC3, 6, 12: clinical competence in the third, sixth, and twelfth months; CS3, 6, 12: clinical stress in the third, sixth, and twelfth months; ITL3, 6, 12: intention to leave in the third, sixth, and twelfth months. *P < .05; **P < .001.
